# Common and unique menopause experiences among autistic and non-autistic people: A qualitative study

**DOI:** 10.1177/13591053251316500

**Published:** 2025-02-15

**Authors:** Martha A Piper, Rebecca A Charlton

**Affiliations:** Department of Psychology, Goldsmiths University of London, UK

**Keywords:** autistic, knowledge, menopause, non-autistic, qualitative analysis, support

## Abstract

Autistic people face both similar challenges to non-autistic people as they navigate menopause and additional unique challenges. Semi-structured interviews with 15 autistic and 14 non-autistic adults (assigned female at birth), explored experiences of menopause. Thematic analysis was carried out for the autistic and non-autistic groups separately. Analysis yielded four overarching themes: information about menopause, experiences of menopause, medical support for menopause and backdrop to the menopause. Each of these contained subthemes which indicated both shared and unique experiences between the groups. Both groups reported a lack of information about menopause, endured negative psychological changes during menopause and experienced menopause alongside other important life events. Autistic people faced unique challenges during menopause, including medical professionals not accommodating autistic differences, uncertainty-induced anxiety and the lifelong impact of living without an autism diagnosis. This study highlights the need for tailored care for this group during the menopause transition.

## Introduction

Autism is a developmental condition characterised by restrictive or repetitive behavioural patterns, social communication difficulties and sensory sensitivity (American Psychiatric Association, 2022). Historically autism research has focused on childhood and there have been few studies in midlife or later-life, with just 0.4% of autism studies in the past decade focusing people aged 50 or older (Mason et al., 2022). There is also a lack of autism research into people assigned female at birth (‘women’^1^), with just 10 women for every 44 men participating in autism research (Lai and Baron-Cohen, 2015; Loomes et al., 2017). These imbalances mean that research into female-specific issues among autistic people, such as reproductive transitions, is lacking (Hoyt and Falconi, 2015).

Given the lack of older people and women in autism research, it is unsurprising that there have been few studies investigating how autistic people expereince menopause (Steward et al., 2018). Menopause is the termination of menstrual cycles due to hormonal changes associated with the loss of reproductive activity (Giannini et al., 2021). The physical symptoms of menopause include hot flushes, muscle aches, headaches and heart palpitations, but mood disorders are also common (Harlow et al., 2012; Sturdee et al., 2017). Research suggests that autistic people are especially impacted by hormonal fluctuations for example during menstruation (Obaydi and Puri, 2008; Steward et al., 2018). Autistic people report distressing symptoms during menstruation which are distinct from those experienced by non-autistic people, including sensory sensitivities and difficulties regulating emotions and behaviour (Steward et al., 2018). Therefore, it is unsurprising that other periods of hormonal changes (such as menopause) may be particularly challenging for autistic people. Although we do not fully understand why hormonal changes may be especially impactful for autistic people, common co-occurring conditions may be important. For example, the sensory sensitivities autistic people frequently experience may exacerbate menopause symptoms (Hoffman et al., 2023). Qualitative studies report that menopause amplified autistic presentation (including sensory sensitivities) and introduced new difficulties which participants’ lifelong coping strategies could no longer remedy (Karavidas and de Visser, 2021; Moseley et al., 2021b). Common, pre-existing conditions such as depression may also cause additional difficulties for autistic people. One study suggests that autistic people are four-times more likely to experience depression compared to non-autistic people (Hudson et al., 2018). Since prior history of depression increases risk of depression during menopause, and depression during menopause is associated with more distressing experiences, autistic people may be at risk for menopause difficulties (Bromberger et al., 2015; Clayton and Ninan, 2010; Maki et al., 2019). This is supported by a previous study in which autistic people reported increased depression and anxiety, and more panic attacks during menopause compared to pre-menopause (Moseley et al., 2021b).

Pre-existing conditions may influence attitudes and expectations of health professionals, as well as causing diagnostic overshadowing where symptoms are incorrectly associated with a pre-existing condition or autism diagnosis (Mason et al., 2019; Moseley et al., 2021a). Autistic people often report poor relationships with health professionals, which may reduce the likelihood of seeking medical support (Mason et al., 2019; Moseley et al., 2021a). When autistic people speak to medical professionals they face difficulties being understood, as they may not report symptoms in the same way as non-autistic patients (Moseley et al., 2021a). It is worth noting that non-autistic menopausal people also report poor interactions with medical professionals (Eriksen et al., 2023). Although over a third of menopausal people contact their health provider, many report that symptoms were not investigated and treatments not offered (Cumming et al., 2015; Newson, 2016; Nuffield Health, 2017). These statistics indicate that despite negative menopausal experiences being common, they remain poorly treated. (It is important to note that current treatments do not work for or are not acceptable to everyone experiencing symptoms). Autistic people may be particularly at risk for poor experiences with healthcare professionals during menopause due to the intersection of menopause and autism.

Results suggest that autistic women find the menopause distressing although no qualitative study has yet compared menopausal experiences of autistic and non-autistic people. To our knowledge only one quantitative study has compared autistic and non-autistic menopause experiences. Autistic people reported more physical and psychological complaints compared to non-autistic people, but no differences were observed in reported urogenital complaints (Groenman et al., 2022). Whether the severity or prevalence of experiences is similar or different among autistic and non-autistic people requires further exploration. The present study uses semi-structured interviews to explore the experience of menopause for both autistic and non-autistic people so common and unique experiences can be explored.

## Methods

### Design

This study received ethical approval from the Goldsmiths, University of London Research Ethics Committee. The study was advertised by word of mouth, through existing participant databases, and social media and described as ‘exploring autistic and non-autistic people’s experiences of menopause’. After participants contacted the research team they were provided with an information sheet, a data protection document and consent form; and were given time to ask questions and decide whether or not to participate. To reduce geographical limitations and barriers to participation, participants could complete the interview in person, via video, voice call or email. Semi-structured interview questions were shared with participants in advance. The loss of spontaneity this introduced was outweighed by the benefit of mitigating uncertainty-induced anxiety around the interview, which is particularly common in autistic people (Jenkinson et al., 2020). Participants gave informed consent by signing the consent form or giving verbal consent at the start of the interview. Interviews were conducted between April and September 2023. The video and audio call interviews were transcribed using Microsoft Teams. The transcripts were edited to correct for errors in transcription and to anonymise the data for use in the analysis. Participants were sent a debrief document following their participation and were offered a £20 gift voucher as thanks for participating.

### Participants

There were no exclusion criteria regarding gender, although participants had to have experience of menstruation and menopause. All participants were cis females. Autistic individuals were included if they self-identified as autistic or had a formal diagnosis of autism. Self-identified autistic people completed the Autism-Spectrum Quotient (AQ), where a score above 32 indicates clinically significant autistic traits (Baron-Cohen et al., 2001). Inclusion of self-identified autistic people is considered good practice as rates of diagnosis are often low in this age-range and for women (O’Nions et al., 2023). Fifteen autistic people completed the interview (3 via email; 12 via video call), 14 had a formal autism diagnosis and one person self-identified (AQ score = 33). Fourteen non-autistic participants completed the interviews (4 via email; 10 via video call). See Table 1 for demographic information. To allow attribution of quotes to individuals, each participant was given a pseudonym either beginning with A (for autistic participants) or N (for non-autistic participants).

**Table 1.** Demographic participant information.

Pseudonym	Age at interview	Age at menarche	Age at last period	HRT	Autism diagnosis	Age at autism diagnosis
Ava	46	12	45	For a period, not currently	Diagnosis	41
Amelia	52	11	51	No	Diagnosis	45
Abigail	53	13	53 (3 months previous)	For a period, not currently	Diagnosis	53
Aurora	49		46	Yes	Diagnosis	42
Audrey	47	15	Menstruating	No	Diagnosis	42
Anna	55	12	54	No	Diagnosis	47
Alice	50	15	Menstruating	Yes	Diagnosis	45
Athena	53	11	52	No	Diagnosis	45
Alex	66	14	48	Yes	Diagnosis	59 (suspected since 45)
Amanda	44	12	Menstruating	Yes	Diagnosis	37
Artemis	54	13	46	No	Diagnosis	53
Amy	57	13	51	No	Self-identified	45
Acacia	49	11	46	Yes	Diagnosis	43
Ariel	52	17	47	No	Diagnosis	37
Amira	57	13	48	Yes	Diagnosis	57
Nora	67	13	59	Yes	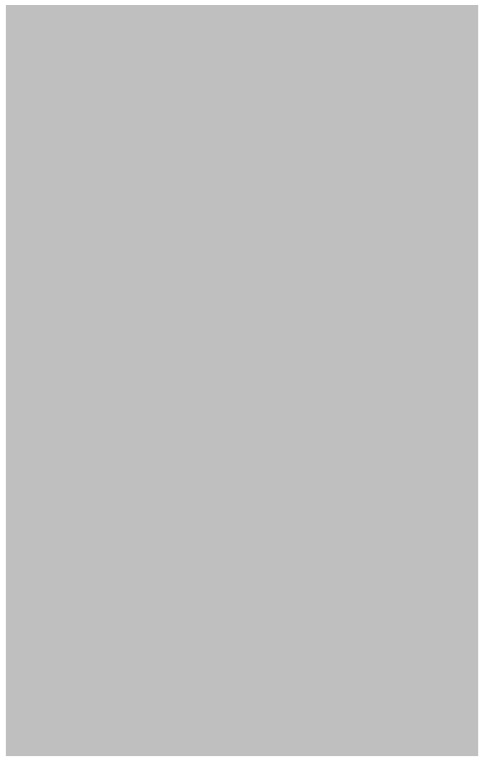	
Norma	52	12	49	For a period, not currently		
Nicky	45	13	Menstruating, irregular	Yes		
Nicole	61	13	55	No		
Naomi	55	12	52	No		
Natalie	58	10	57	For a period, not currently		
Natalia	59	14	49	Yes		
Nina	57	10	52	Yes		
Nia	51	13	42	No		
Nancy	55	15	51	No		
Natasha	53	11	52	No		
Nadine	49	13	41	No		
Nadia	67	14	48 (medically induced)	No		
Niamh	84	11 or 12	50	No		

### Data collection

Interviews asked for background information about menstruation, menopause and access to support. Questions asked about both positive and negative experiences of physical and psychological menopause symptoms (see Table 2). The questions were concise and avoided vague language. The semi-structured nature of the interviews allowed follow-up questions and for individuals to discuss other experiences.

**Table 2.** Semi-structured interview questions.

1. Name:
2. How old are you?
3. What is your gender, and is this different from the sex you were assigned at birth?
4. Which country do you live in? Is this different to where you were born?
5. Are you employed at the moment?
6. *Do you have an autism diagnosis?*
*If yes, how old were you when your autism was diagnosed?*
*Do you have any other neurodiversity or diagnoses that you think we should know about?*
I will now ask you about your experience of menstruation.
7. How old were you when you had your first period?
8. When you were menstruating, what were your periods generally like?
(Regularity, discomfort)
9. When was your last period?
10. How long have you been going through the menopause for?
11. What led you to believe you had started the menopause?
I will now ask you about the mental and physical experiences you had during the menopause.
12. Starting with the physical, which physical changes did you notice, if any?
a. Were there physical aspects of life which became harder?
b. Were there physical aspects of life which became easier?
13. Moving onto the psychological experiences, did you notice any differences during the menopause?
a. Were there positive psychological changes during the menopause?
b. Were there negative psychological changes during the menopause?
14. Have you considered or received any medical support for menopause related symptoms, such as HRT, holistic natural remedies or self-care activities?
a. Why/why not?
b. If yes, have these helped?
c. How many times have you tried to find help?
15. Have you considered or received any social support for menopause related symptoms?
a. Why/why not?
b. If yes, have these helped?
16. What advice would you give to someone who was about to go through the menopause?
a. *Would this advice be different if you were speaking to an autistic person?*
17. Where would you look for information about the menopause?
a. Did you access information before starting menopause?
b. Did you feel prepared for what was to come?
18. Are there any changes that you experienced during the menopause that you have since realised were symptoms?
19. Is there anything else you’d like to mention or ask? Is there anything we may have missed?

Questions in italics are those only given to autistic participants.

### Data analysis

A qualitative approach allows for rich, nuanced data which considers the wider context in which the experience occurs. This research adopted a constructivist epistemology, which assumes that reality is subjective and constructed by each individual through culture, language and behaviour. This was coupled with a relativist ontology, which aims to explore a particular experience and how it is shaped by individual circumstances, rather than discover objective, generalisable truths. This nuanced, in-depth approach is appropriate to explore the experiences of autistic and non-autistic women during menopause in the context of their individual circumstances and perspectives.

Thematic analysis was carried out using the 6-stage framework laid out by Braun and Clarke (2006, 2019). The researchers took an inductive approach, developing themes and codes which reflect both surface meaning and latent underpinnings of experiences (Fereday and Muir-Cochrane, 2006; Grogan and Mechan, 2017). One researcher independently immersed herself in the data, generated and refined codes and created themes and subthemes. Themes were discussed with and re-examined by the other researcher to enhance trustworthiness of the analysis. Following the 29 interviews, there were no new themes being generated and it was deemed that data saturation had been reached. The themes for autistic and non-autistic people were generated separately and the results were compared. Quotes were selected to illustrate themes and subthemes.

### Reflexivity

An important consideration throughout the analysis was the bias introduced by the interviewers’ expectations and experiences on their interpretation of the data. The research team were an academic researcher and postgraduate student. Both members of the team have a background in research working with older people and autistic people examining variables that impact well-being. Both team members were female, with some having and others not having experience of the menopause. Attitudes to the menopause were mixed across the team with one person expecting menopause to have a negative impact and the other person expecting a mixture of experiences to be reported. Interviewer bias was managed by the inclusion of questions which asked specifically about both positive and negative changes experienced. Neither of the researchers involved in this study were autistic, although the project was discussed with the wider research team which includes autistic researchers. The researchers aimed to limit their impact by using open-ended follow-up questions which did not anticipate a certain answer.

## Results

**Figure 1.**
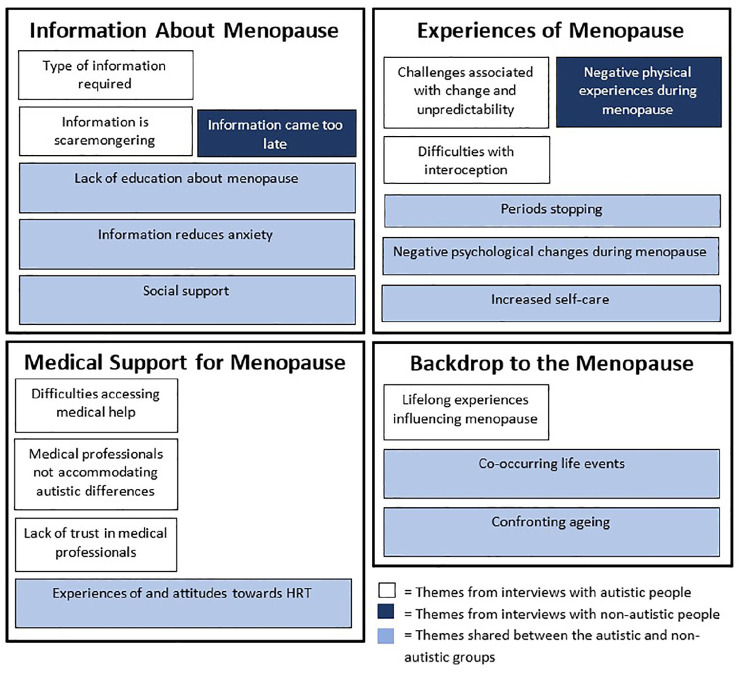
Table of themes and subthemes for both groups of participants.

Although we performed thematic analysis separately for the autistic and non-autistic groups, subthemes clustered into four common themes: (1) *Information About Menopause*, (2) *Experiences of Menopause*, (3) *Medical Support for Menopause* and (4) *Backdrop to Menopause*. These four themes are each comprised of subthemes, which are either unique to one group or common to both the autistic and non-autistic groups. The results are summarised in Figure 1.

### Information about menopause

This theme reflects people’s access to information about menopause and whether that information was seen as helpful or detrimental to their experience.

#### Lack of education about menopause (both groups)

People from both groups failed to recognise the first signs of menopause because they lacked awareness of what it might involve. People explained that they held false beliefs about menopause, such as that it ‘*was something that happened in your 50s*’ (Ava). The little knowledge people did have about menopause was anecdotal, and rarely matched their own symptoms. When people did educate themselves, they ‘*would rather have read that before the menopause*’ (Nina) to be more prepared for what was to come.

#### Information reduces anxiety (both groups)

People from both groups explained that not knowing about possible menopause symptoms meant they felt confused and scared when first experiencing them. Nancy explained, ‘*the brain fog and the irritability and the restlessness at night I didn’t know what I was going through*’ and Amira recalled ‘*it felt like my brain had gone offline for a few seconds. It felt extremely scary*’. Information about menopause made people feel ‘*reassured because I understand what’s going on*’ (Ava). People said the increased media coverage, not only reduced anxiety but because ‘*menopause is becoming less of a kind of insult*’ (Alice). This reduced stigma around menopause positively feeds back into education, as it reduces barriers to getting information, such as embarrassment.

#### Social support (both groups)

Both autistic and non-autistic people spoke about the role of social support during menopause, but need for and access to this support varied between the groups. Non-autistic people spoke about the importance of their ‘*very supportiv*e’ (Nancy) friendships which had ‘*an awareness and an understanding of what we’re going through*’ (Natasha). In comparison, many autistic people felt their friendships did ‘*not go very deep*’ (Artemis) which was exacerbated when psychological symptoms of menopause meant they ‘*disassociated, withdrew from people, couldn’t communicate*’ (Abigail). There were exceptions to this rule, some autistic people felt they had ‘*a very close friend group*’ (Amy) and others recalled ‘*really open*’ (Amelia) discussions about menopause with their mothers. Some non-autistic people felt they lacked social support, but this was for reasons such as ‘*lots of my friends hadn’t started the menopause when I did*’ (Nadine), rather than the feelings of isolation commonly described by autistic people.

Friendships were important for information sharing about menopause, with several non-autistic people advising ‘*talk to your mates, [be]cause I’ve learned so much from other people*’ (Nia). Conversely, autistic people described how a lack of social support reduced the information they naturally learnt about menopause. Ava described ‘*not having the social contacts to discuss things with*’. Instead, autistic people said they would go to ‘*social media first*’ (Acacia) for information because they felt they were ‘*not in a place to see real people*’ (Amira).

#### Type of information required (autistic group)

Autistic people spoke about needing scientific information about menopause delivered in a direct way. Artemis said that she and her autistic peers seemed to ‘*strongly relate to information, not so much emotion*’. In some cases, this influenced the kind of support they needed for menopause, with Abigail explaining her need for ‘*black and white*’ fact-based information rather than emotional support from peers.

#### Information is scaremongering (autistic group)

Although people recognised the importance of education about menopause, several autistic people commented that certain types of information were ‘*scaremongery*’ (Abigail). Athena explained that too much emphasis on the difficulties of menopause ‘*encourages an expectation of problems rather than freedoms*’ and Anna said that symptoms ‘*might or might not [happen]. There is no point in alarming someone*’. Autistic people spoke about how sensationalism of the menopause in the media may feed into the view that women are ‘*at the mercy of our hormones*’ (Athena) which they felt was disempowering.

#### Information came too late (non-autistic group)

Non-autistic people said that they only recognised their symptoms as menopause in hindsight. People explained that ‘*[menopause] is happening before you recognise it is happening*’ (Nancy) which meant they ‘*didn’t even look up what menopause was until my periods stopped… and it was all over*’ (Niamh). People described dealing with symptoms individually; it was only when they looked back that people were able to ‘*paint the picture*’ (Natasha) and realise they were all linked. Nancy explained ‘*hindsight is a great tool for recognising menopause*’. This retrospective re-evaluation of events was not described by the autistic people.

### Experiences of menopause

This theme reflects the physical and psychological changes during menopause.

#### Periods stopping (both groups)

Experiences of periods were varied, with some people having ‘*very heavy and very long [periods with] bad stomach cramps*’ *(*Norma) which ‘*crippled*’ them (Amira). Others felt periods were ‘*very easy*’ (Nadine) or ‘*not much of an event*’ (Ariel). Some people explained that their periods became heavier before menopause with periods being described as ‘*horrendous*’ (Abigail). Autistic people struggled with the unpredictability of their periods, which made it difficult to plan. Several people expressed relief at their periods stopping during menopause, saying ‘*periods stopping was fantastic*’ (Naomi) and that it had ‘*definitely made my life easier*’ (Ava). Amy explained that her periods stopping had enabled her to ‘*reach a level of stability emotionally and physically*’ which was ‘*absolutely bloody marvellous*’.

#### Negative psychological changes during menopause (both groups)

People from both groups experienced a range of negative psychological changes during menopause, from anger to social withdrawal. Amy described ‘*the most intense rage you can imagine*’ and Nia experienced ‘*massive mood swings*’ which made her feel like she ‘*could kill people quite easily*’. Others experienced low mood and lack of motivation, with Natalie explaining, ‘*I had to push myself to do ordinary things like empty the dishwasher*’. Amelia’s low mood and lack of motivation became ‘*a vicious cycle because you just feel miserable about yourself*’. Linked to this, people spoke about reduced self-esteem during menopause. Nina ‘*started doubting myself more*’ and Aurora said her ‘*self-image and self-confidence dropped to almost nothing*’. Another common psychological change was irritability and tearfulness, with people describing becoming ‘*easy to trigger*’ (Nicky), getting ‘*tearful over ridiculous things*’ (Natalia) and feeling like ‘*crying most of the time*’ (Amira). Several people also experienced increased anxiety during menopause. Nadia, who had suffered with panic disorder in the past, said ‘*panic attacks were more frequent*’ and were often ‘*simultaneous with the hot flash*’. Some people’s anxiety had knock-on effects for their sleep. Aurora recalled ‘*waking early with extreme panic*’ and Natasha recalled waking at ‘*3 or 4 o’clock in the morning and … not be able to stop thinking about ridiculous things*’. Some people felt they had become socially withdrawn during menopause. Natalie explained ‘*I’m not social anymore*…*If I can stay away from people I will*’ and Abigail ‘*just disassociated, withdrew from people, couldn’t communicate, didn’t want to*’.

#### Increased self-care (both groups)

Both autistic and non-autistic people spoke about creating new health-promoting habits during menopause. Several people chose to prioritise exercise in an effort to combat their physical and psychological symptoms. Natalia ‘*tried to up the exercise a bit more and the achiness, which was my worst symptom, seems to have eased*’ and Aurora ‘*was feeling a little wobbly with my mental health and started seeing a personal trainer*’. Some people said they focused on strength-based exercise, as they felt ‘*the need to be stronger*’ (Nancy) to counteract feelings of ageing during menopause. Some people spoke about the importance of changing their diets to manage menopause symptoms. Ava described how cutting back on alcohol, caffeine and sugar and taking herbal supplements meant most of her ‘*menopause symptoms have disappeared*’ and Niamh had ‘*focused on healthful foods and enjoyable exercise*’. Another important lifestyle change for both groups was slowing down and tuning into their bodies. When asked what advice she’d give to a pre-menopausal person, Amelia advised, ‘*just be aware that it is a quietening time and get a bit in tune with your body*’. Some people found it difficult to prioritise their own needs as they had other caring responsibilities. Natasha was used to ‘*keeping going when you’re tired or when other stuff is going on*’ which meant she rarely took time out for herself.

#### Challenges associated with change and unpredictability (autistic group)

Autistic people spoke about struggling with the changes happening in their bodies during menopause. Amy described ‘*feeling quite a bit of grief about*…*losing something* that is *fundamental about being female*’ and Amira felt ‘*terrified because it was a change in me*…*I didn’t even know who I was*’. These changes happened in unpredictable ways and people described feeling ‘*hacked off that I can’t plan*’ (Acacia), because ‘*change is not something I can deal with very well*’ (Artermis). Some people recalled struggling with similar unpredictable change during puberty or pregnancy. To manage menopause more smoothly, Abigail advised autistic people ‘*don’t be scared of it*…*put strategies in place so that it’s a smooth transition*’.

#### Difficulties with interoception (autistic group)

Autistic people also described difficulties with interpreting unpredictable changes in their bodies due to poor bodily awareness. Audrey said, ‘*I struggle sometimes to be aware of what I’m feeling*’ and Acacia explained that ‘*being autistic means I don’t always pick up on things*’ that were changing in her body. Amira explained that she was hypersensitive to some sensations and hyposensitive to others, which meant she didn’t trust her bodily signals. As a result, she ‘*operated as a head prior to menopause*’ and found it difficult when menopause ‘*forced me to look at the whole of my body*’. Late diagnosed autistic people commented that they had experienced the world for so long without recognition of their autistic differences. This led them to disbelieve bodily signals, which autistic people felt would ‘*gaslight*’ (Amy) them. Advice for other autistic people included ‘*know what the physical effects are*’ (Abigail) and to note down all bodily changes in a journal to help promote bodily awareness and make sense of menopause symptoms over time.

#### Negative physical experiences during menopause (non-autistic group)

Non-autistic people tended to focus on negative physical changes and speak about the adverse effects menopause had on their lives. Even when asked about the positive changes they had experienced, very few were reported. People experienced a wide range of menopause symptoms but the physical symptom most often reported was weight gain. Nina explained ‘*I’m not eating any more, but I feel weightier*’ and Natalie said she had ‘*put on a lot of belly fat*’. It had become ‘*very, very hard to lose weight now*’ (Natalia) and exercise ‘*doesn’t seem to make any difference at all*’ *(Natasha)*. Although autistic people did not spontaneously report many physical symptoms, they were described when directly questioned.

### Medical support for menopause

This theme reflects people’s experiences of accessing medical support for menopause, including interactions with medical professionals and experiences with HRT (Hormone Replacement Therapy).

#### Experiences of and attitudes towards HRT (both groups)

Attitudes towards HRT were mixed in both groups. Six of the 14 non-autistic women and eight of the 15 autistic women had tried HRT at some point. Those who did not take HRT gave various reasons such as existing health contraindications, ‘*the [ageist and sexist] stereotypes attached to* [*HRT*]’ (Ariel), being ‘*very anxious about…taking new medication*’ (Ava) and preferring ‘*to work through [psychological issues] in other ways*’ (Nancy). Others had more positive attitudes to HRT, with Natalia reasoning that ‘*the side effects of having menopause are worse than the potential side effects of having HRT*’.

The experiences of taking HRT were also mixed in both groups. Some people praised HRT, with Amira saying it ‘*enabled me to build my life back again*’. Some people had neutral experiences of HRT, saying ‘*it didn’t make a difference*’ (Abigail) to menopause symptoms. Others had very negative experiences, with Amy explaining that since going on HRT for osteoporosis, she had experienced ‘*a roller coaster of depression and anxiety*’ which was so bad that she would rather ‘*have my bones crumble*’. Some autistic people struggled with HRT for other reasons. For example, Alice felt the mixed administration of gels and tablets was ‘*too complicated*’ to get her head around and said that the ‘*sticky marks*’ from HRT patches caused her sensory discomfort.

#### Difficulties accessing medical help (autistic group)

Many of the autistic people encountered barriers to accessing medical help for menopause. They described their GPs (General Practitioner Doctors) as ‘*unresponsive*’ (Ariel) and ‘*overwhelmed*’ (Acacia) due to Covid-19. Even once she had seen her GP, Amira had ‘*fought and fought*’ to see a menopause specialist to help with her HRT dosage. Similarly, Aurora ‘*went to the GP three times before I was permitted to try HRT*’ and in the meantime paid for a private scan because she was so concerned about her heavy bleeding.

#### Medical professionals not accommodating autistic differences (autistic group)

Autistic people felt that doctors needed to take a different approach with autistic patients. They explained that autistic people tended to ‘*look at things quite factually*’ (Artemis) and often created lists of their symptoms to present their doctors. This was met with dismissive comments including ‘*Oh I’ve got no time to look through all these*’ (Aurora) which left people feeling ignored by their doctors. Amanda felt that she wasn’t taken seriously by her doctor, as ‘*autistic people are automatically on the hypochondriac list at the GP*’. In other cases, the autistic tendency towards information meant doctors made ‘*an assumption that I knew what was going on*’ (Ava), which led to confusion about how to administer prescribed HRT. The lack of accommodations made by GPs led some people to seek help elsewhere, with Acacia paying for a private GP consultation because she felt ‘*an NHS [National Health Service] one just wouldn’t listen to me*’. The private doctor ‘*didn’t dismiss any of my symptoms*’ (Acacia) and made accommodations for her autistic differences, such as the option to meet online, medication delivered to her home and a treatment plan in writing.

#### Lack of trust in medical professionals (autistic group)

Many autistic people had difficulty trusting the advice of medical professionals. This may have been influenced by lifelong experiences of living without an autism diagnosis. Amira had struggled with autistic overwhelm her whole life but, not having an autism diagnosis, was given a series of mental health medications which did not work. This made her feel ‘*like a walking guinea pig*’ and ‘*inadequate because it wasn’t working*’. This lack of trust persisted after their autism was diagnosed. Ariel felt that her autism diagnosis had been ‘*used in a very reductively simplistic and not very honest way*’ by medical professionals. Acacia commented on the high rates of anti-depressant prescriptions saying, ‘*antidepressants do a lot of heavy lifting in this space [but they] don’t work if you*’*re not depressed*’. Others were sceptical about doctors’ motives, with Anna saying her GP encouraged women to have hysterectomies as it made them ‘*tidier, easier to manage and more available for work and sex*’.

### Backdrop to the menopause

This theme reflects the wider context in which people experienced the menopause transition and how it impacted their experience of menopause.

#### Co-occurring life events (both groups)

People from both groups explained that it was difficult to know which changes were due to menopause and which were due to other co-occurring life events. Some people attributed their physical changes, such as weight gain, to lifestyle changes rather than menopause, with changes in their home life being a major influence, ‘*a complete change in my environment… meat, ice cream, potato chips, a sedentary job*’ (Niamh). Similarly, Alex expressed confusion about whether her lack of energy was due to menopause or another condition, questioning ‘*What is [due to] menopause? What is chronic fatigue? What is fibromyalgia? What is just depression?*’. Other people explained it was difficult to attribute psychological changes to menopause, when there was so much change going on in their personal lives.

#### Confronting ageing (both groups)

People from both groups said menopause had made them reflect on ageing. Some expressed sadness that they were no longer able to have children, saying it was ‘*distressing to suddenly realise your reproductive life is just over*’ (Amelia). Similarly, others had ‘*a feeling of lost time*’ (Ava) as if ‘*you haven’t reached the milestones in life that you wanted to*’ (Anna). Some people described feeling ‘*like I’ve aged suddenly really quickly*’ (Norma) and as if ‘*I’ve lost my pizazz*’ (Alex). This impacted their self-esteem, with Aurora saying she felt she became invisible and Anna worrying about attracting a partner later in life. However, some people felt less negatively about ageing, describing it as ‘*natural*’ (Nancy), ‘*normal*’ (Nia) or even ‘*a cause for celebration!*’ (Athena).

#### Lifelong experiences influencing menopause (autistic group)

Several autistic people explained their lifelong feeling of not fitting extended into menopause. Artemis said she was ‘*quite an expert on what it feels to be not normal*’. Autistic people described a history of masking their autistic traits for fear of drawing attention to themselves: ‘*you’re constantly trying to fit in with how society wants you to be… your true self isn’t going to be accepted*’ (Amelia). This carried on during the menopause, with Amira creating ‘*a menopause mask on top of the autistic one*’. This meant that autistic people tended not to ask for help for their menopause symptoms. Acacia felt ‘*if I wasn’t autistic, I would have gone to the doctor and said, can you help me?*’.

## Discussion

The qualitative analysis identified four themes describing common and unique experiences of menopause for autistic and non-autistic people. Common experiences were described in terms of information, experiencing symptoms and backdrop to menopause, whereas there were more unique experiences around medical support.

A common theme related to the need for and availability of information. Social support was identified as an important route to accessing information during menopause but attitudes to discussing menopause with peers varied between the groups. Non-autistic people described the importance of discussing menopause with their family and friends for learning about symptoms and management options. In contrast, most autistic people did not rely on friends or family, but sought information from social media. This is consistent with existing research which found that autistic people have smaller social circles which further reduce with age (Chan et al., 2023; Charlton et al., 2022). Autistic people described that their reduced social support limited information sharing through social learning, as they had fewer conversations with peers about menopause. Autistic people were hesitant to discuss menopause with the social connections they did have for fear of oversharing, as they did not know what was appropriate to discuss in terms of menopause symptoms. This is in keeping with previous findings that autistic people used self-censorship when discussing menopause, perhaps related to a tendency to mask autistic differences during social situations (Brady et al., 2024). Some people described withdrawing from social connections during menopause, due to increased communication difficulties and mental health symptoms. This is consistent with Moseley et al.’s (2021b) study which found that autism-related communication differences became heightened during menopause and may be a barrier to accessing support. Improving social support for autistic people before and during menopause is likely to be beneficial for improving knowledge and reducing stigma (Brady et al., 2024).

There were also differences between the groups in attitudes to menopause in society. Non-autistic people described how menopause had lost its taboo since becoming more prevalent in the media. However, autistic people reported feelings of stigma associated with menopause. This is consistent with previous results suggesting that autistic people were apprehensive when speaking about menopause with their friends (Brady et al., 2024; Moseley et al., 2021b) and uncomfortable discussing menopause with medical professionals (de Visser et al., 2024). Attitudes towards menopause may be important, as some studies suggest that those with a negative attitude towards menopause report more menopause symptoms (Ayers et al., 2010). This may represent ‘a double whammy of taboos, autism and menopause’ preventing autistic people from discussing symptoms and having potential negative impacts on information and advice seeking (Brady et al., 2024: 1411).

In terms of menopause symptoms, both groups discussed their experiences in predominantly negative terms, describing how menopause worsened their quality of life. The most prevalent physical symptoms were hot flushes, night sweats, insomnia, reduced libido, weight gain and fatigue which were spontaneously reported by non-autistic people. Autistic people tended to report psychological symptoms over physical symptoms. However, when asked directly whether a range of physical symptoms occurred, they were reported at similar rates in both groups (data not shown). In addition, unique difficulties with interoception were described by autistic people, in keeping with some previous findings (Brady et al., 2024; Moseley et al., 2021b). Autistic people reported that interoception difficulties increased struggles during menopause as they didn’t trust their bodily signals. Some autistic people described how learning about the common symptoms of menopause helped them look out for any changes and foster an awareness of their bodies more generally, with some crediting menopause with triggering a newfound appreciation for and attunement with their bodies.

Psychological changes were common but varied between individuals, reflecting the heterogeneity of menopause among all people. Autistic people tended to emphasise psychological changes when asked about their experience of menopause (compared to non-autistic people), indicating a substantial impact on their lives. This is similar to the findings of Moseley et al.’s (2021b) study in which autistic people’s mental health declined during menopause and continued to worsen as they tended not to seek help. This is reflected in our subtheme *lifelong experiences influencing menopause* where autistic people described how their tendency to mask their autistic characteristics and ‘act normal’ had carried on into menopause, leading them to cope with menopause symptoms rather than asking for help. Some people attributed psychological difficulties to a loss of control over circumstances and emotions, which is linked to our subtheme of *challenges associated with change and unpredictability*. Importantly, many of the autistic people (9/15) had been diagnosed with a mental health problem in the past (compared 2/14 of the non-autistic people). (However, we note that several autistic people felt their pre-existing mental health problems were in fact misdiagnoses of their autistic traits before they received an autism diagnosis.) Pre-existing depression has been found to be a risk factor for depression during menopause (Alblooshi et al., 2023). Although previous studies have found that menopause may amplify autistic traits (increased sensory sensitivities and meltdowns), this was not identified as a theme in the current analysis (Moseley et al., 2021a, 2021b). Previous studies have also identified menopause as a trigger for diagnosis (Moseley et al., 2021b) and this was not replicated in the current study. In the current study only four of the 15 autistic people were diagnosed during menopause and none were reported as triggered by menopause-related changes. Menopause may cause extreme psychological changes for autistic people, but it is not clear whether this is due to neurodiversity-related differences, existing mental health problems or difficulties accessing social and medical support.

Although autistic and non-autistic people had similar experiences and attitudes to HRT, autistic people reported more challenges in accessing medical support and interacting with health care professionals. This may be surprising since most people were accessing medical support via the same National Health Service in the UK. However, this finding is in keeping with research suggesting that autistic people find the process of accessing appointments overwhelming, often related to needing to speak on the phone (Brady et al., 2024). Furthermore, getting an appointment may be more effortful for autistic people due to anxiety and past negative experiences with medical professionals (Brady et al., 2024; Pohl et al., 2020). Autistic people commonly reported that medical professionals did not accommodate autistic communication differences and described feeling dismissed or ignored. This is in keeping with research which found that autistic people are often anxious about what information to share during medical consultations and may prefer to use written communication (Mason et al., 2019; Pohl et al., 2020). However, medical professionals rarely accommodate their communication preferences, which may lead to incorrect assumptions about autistic patients’ symptoms and needs (de Visser et al., 2024; Nicolaidis et al., 2015). Difficulties describing bodily signals may contribute to symptoms being dismissed (Brady et al., 2024). Positive experiences did occur, but often in private medical appointments where autistic people felt they had more time and control over the appointment. These negative experiences indicate that improving awareness of autism communication differences among health professionals remains a training need (Mason et al., 2019).

Although people associated mainly negative physical and psychological changes with menopause, some also commented that things improved after menopause. For example, people from both groups described how menopause helped them slow down and increased their self-compassion. Some people commented they had adopted healthy habits to manage menopause symptoms and carried these on after menopause, which had a positive impact. Others described a newfound sense of self-esteem and confidence after menopause, as well as feeling joy about getting older.

To our knowledge only one other study has compared the experiences of autistic and non-autistic people, which limits available comparisons (Groenman et al., 2022; however see Brady et al., 2024; Karavidas and de Visser, 2021; Moseley et al., 2021a, 2021b for autistic-only experiences). Therefore, results should be interpreted with caution and within the bounds of certain limitations. Participants were recruited through word of mouth, databases of study participants and social media. This indicates that participants were cognitively able, independent and that some had taken part in previous research studies. Therefore, experiences may not reflect the experiences of the wider autistic or non-autistic population. It is worth noting that where people participated in previous studies, these were not on the topic of menopause and therefore there should be limited bias by recruiting through those channels. Non-autistic people were not asked to complete the AQ, and therefore there is a risk that this group could include undiagnosed (and unaware) autistic people. It is worth noting that interviews were conducted through different media including via email. The aim of this was to increase access and results do not suggest systematic differences in experience related to the interview type.

Results in this study indicate the presence of both common and unique menopause experiences for autistic and non-autistic people. Many experiences were similar between the groups, with evidence for individual differences in experiences and symptoms. Subtle differences were also apparent, with some common themes being expressed differently for autistic and non-autistic people. Importantly, unique experiences were also reported particularly for the autistic people. It is important for healthcare professionals to both accommodate autistic communication differences and allow for differences in the way symptoms are described to ensure appropriate treatment and support. Further studies examining experiences in autistic and other neurodivergent groups are vital to support wellbeing through menopause.

## Notes

1. We use the term ‘women’ in this paper to indicate people assigned as biologically female at birth, while acknowledging that some people assigned female at birth may not identify as ‘women’. Wherever possible we use the term ‘people’.
